# Polyarthritis Caused by Methimazole in Two Japanese Patients with Graves’ Disease

**DOI:** 10.4274/Jcrpe.1055

**Published:** 2013-12-12

**Authors:** Hiroko Nihei, Hidenori Tada, Yuki Naruse, Masako Izawa, Manji Kato, Hiroaki Okuno, Akie Nakamura, Katsura Ishizu, Takashi Hamajima, Toshihiro Tajima

**Affiliations:** 1 Hokkaido University School of Medicine, Department of Pediatrics, Sapporo, Japan; 2 Aichi Children’s Health and Medical Center, Department of Pediatric Endocrinology and Metabolism, Osakada, Morioka-cho, Obu, Japan; 3 Kami-Iida Daiichi General Hospital, Department of Surgery, Kita-ku, Kamiiida Kita-Machi, Nagoya, Japan; 4 Kuchan Kosei Hospital, Department of Pediatrics, Kuchan-Cyo, Hokkaido, Japan

**Keywords:** Graves’ disease, methimazole, adverse event, polyarthritis

## Abstract

In many countries, methimazole (MMI) therapy is the first-line treatment in children with Graves’ disease (GD). The rate of side effects of antithyroid drugs (ATDs) in children has been reported to range between 6% and 35%. Of these side effects, polyarthritis is uncommon but serious, and can also develop as a part of the antineutrophil cytoplasmic antibody-associated vasculitis that is induced by ATDs. Here, we describe two GD girl patients aged 15 years and 11 years who developed polyarthritis. The onset of polyarthritis in these patients was 24 days and 28 days after the initiation of MMI therapy, respectively. MMI was suspected of causing the polyarthritis in the two patients and was withdrawn. The symptoms of polyarthritis disappeared rapidly following cessation of treatment. Subsequently, one patient was treated with 131I therapy and the other patient was subjected to thyroidectomy. Although it rarely occurs in pediatric GD patients, severe polyarthritis is a serious side effect of MMI and is an indication for prompt cessation of treatment.

**Conflict of interest:**None declared.

## INTRODUCTION

Graves’ disease (GD) is an autoimmune disease caused by thyroid-stimulating autoantibodies and is typically characterized by symptoms such as emotional lability, fatigue, tremor, palpitations, ophthalmopathy, myxedema, and acropachy. Although the primary treatment for children with GD in many countries is antithyroid drugs (ATDs) therapy with methimazole (MMI) and propylthiouracil (PTU) (1,2,3), these drugs have multiple potential side effects (1,4,5). Although MMI has a better overall safety profile than PTU (1,4,5), the adverse effects of this medication range from mild, such as cutaneous reaction and arthralgias, which occur relatively frequently (1-6%), to life-threatening ones, such as agranulocytosis, hepatitis and polyarthritis, which are relatively rare (0.5%-2%) (4,5). Occurrence of ATD-induced arthralgia and arthritis has also been reported in children treated with MMI (6,7,8,9).

Here, we report two pediatric GD patients who developed polyarthritis during treatment with MMI. 

## CASE REPORT

**Case 1**

**Figure 1:**
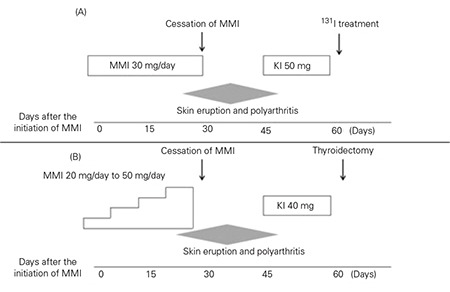
Clinical course of patient 1 (A), clinical course of patient 2 (B).In patient 2, MMI dose was gradually increased from 20 mg/day to50 mg/day. Shaded triangles indicate the course and degree of skineruption and polyarthritis

A 15-year-old Japanese girl was referred to our hospital with symptoms of finger tremor. At this time, she had noticeable goiter. On physical examination, height was 159.4 cm (between 50th and 75th percentiles by Japanese standards for growth). Her pulse was 124 beats/min and her blood pressure was 120/48 mmHg. She had mild exophthalmos. Ultrasonography revealed a diffusely enlarged thyroid gland; its volume was 42.5 mL. Thyroid hormone tests showed that serum free thyroxine (fT4) and triiodothyronine (fT3) were markedly elevated and were 4.29 ng/dL (normal range for fT4, 1.16-1.54 ng/dL) and 32.5 pg/mL (normal range for fT3, 2.61-4.45 ng/dL), respectively, while thyroid stimulating hormone (TSH) was below the normal range (<0.05 mIU/L). TSH receptor antibody (TRAb) test was positive (7.1 IU/L, normal range, <2.0 IU/L). We also determined thyroid-stimulating antibody (TSAb) using porcine thyroid cell cyclic AMP production by a commercial available assay kit (Yamasa, Chosi, Chiba, Japan) according to a previous report (10). TSAb was 488% (normal range <180%). Based on these findings, a diagnosis of GD was made and MMI (30 mg/day) was started. Clinical course is summarized in Figure 1A. 24 days after the initiation of MMI, the patient developed skin eruption and arthralgia involving the hip, shoulder and knee joints bilaterally. While cutaneous reaction and arthralgias were considered to be induced by MMI, these reactions were mild, and treatment was continued with addition of an antihistaminic drug and acetaminophen. However, these medications were ineffective, and arthralgia progressed to involve the wrists, fingers, ankles, and jaw. The degree of pain and swelling and the number of affected joints increased daily, and body temperature increased to 38˚C. At this time, laboratory investigations showed that hemoglobin was 10.9 g/dL, total white blood cell count (WBC) was 8600/μL, platelet count was 26.1x104/μL, and C-reactive protein (CRP) was slightly elevated at 3.41mg/dL (normal range <0.2 mg/dL). Tests for antineutrophil cytoplasmic antibodies (ANCAs) and rheumatoid factor were negative. When polyarthritis worsened, MMI was withdrawn 5 days after the onset of arthralgia and polyarthralgia. The joint swelling and skin eruption gradually disappeared 5 days after the cessation of MMI. For treatment of GD, 50 mg of inorganic iodine was started, and thereafter, the patient was referred to another hospital where one of the authors was working. In Japan, treatment with 131I therapy and thyroidectomy in patients with GD younger than 18 years old is still controversial (11). We therefore consulted with the patient and her parents before deciding on use of 131I therapy. This therapy was effective. After 8 weeks of radiation therapy, levothyroxine was started. In the course of 6 months of follow-up, the goiter disappeared and no arthritis was detected.

**Case 2**

Our second patient was an 11-year-old girl with GD. Her clinical course is summarized in Figure 1B. She was diagnosed as GD at another hospital 40 days prior to referral to the hospital of one of the authors. Her laboratory results and clinical course prior to presentation to our hospital were as follows. She complained of goiter, easy fatigue and palpitation. Physical examination showed a symmetrical, diffusely enlarged thyroid gland with smooth contour and there was no exophthalmos. Her pulse was 136 beats/min with a blood pressure of 135/57 mmHg. Height was 147.3 cm (between the 50th and 75th percentiles by Japanese standards). Her weight was 35.1 kg (between the 10th and 25th percentiles). Laboratory examination showed elevated thyroid hormone levels (fT3, 13.74 pg/mL and fT4, 4.10 ng/dL), low serum TSH levels (<0.03mIU/L) and a positive TRAb test (13.1 IU/mL, normal range, <2.0 IU/L). Ultrasonography of the thyroid showed diffusely enlarged thyroid gland; its volume was 30.5 mL. She was diagnosed as having GD, and treatment with MMI (20 mg, 0.5 mg/kg/day) was initiated, increasing to a maximum dose of 50 mg/day 28 days after initiation. At this time, she complained of pain in the right knee joint and her body temperature increased to 38˚C. She proceeded to develop polyarthritis involving the shoulders, hips, knees, ankles, wrists, fingers and toes, in addition to a skin eruption. At this time, full blood count showed a total WBC level of 6800/µL with a neutrophil level of 3660/µL. Hemoglobin and platelet counts were 10.9 ng/dL and 22.1x104/µL, respectively. Electrolyte and liver transaminase levels were normal and CRP level was 2.41 mg/dL. Based on these findings, polyarthritis was suspected to be associated with MMI. MMI was withdrawn, and 20 mg of inorganic iodine was started. For further evaluation, the patient was referred to the hospital of one of our authors. After cessation of MMI, the polyarthritis improved gradually and body temperature returned to normal, but the left wrist and bilateral knee swellings continued and were painful. The patient also complained of left hip joint pain. There was no skin eruption, and ultrasonography failed to detect synovial effusion in any joint. At admission, laboratory results were as follows: elevated erythrocyte sedimentation rate (ESR) (64 mm/h); elevated anti-nuclear antibody (ANA) 320 (normal range <40); myeloperoxidase (MPO)-ANCA, anti-double-stranded DNA antibodies, rheumatoid factor and anti-cyclic citrullinated peptide antibody were all negative. Based on these findings, we excluded vasculitis associated with ANCAs and other collagen diseases. We carefully explained to the patient and her parents that 131I therapy or thyroidectomy instead of ATD treatment would be a better option for definitive therapy for GD. The parents decided on thyroidectomy. Four months postoperatively, the patient is receiving 100 µg of levothyroxine daily and is euthyroid. 

## DISCUSSION

It is important to distinguish polyarthritis caused by MMI from arthralgia as a minor adverse event occurring during treatment of GD. In the cases we have described here, arthalgia developed to encompass swelling of multiple joints. In addition, serum inflammation tests such as CRP and ESR were elevated. Accordingly, the polyarthritis in both cases was considered to be severe rather than mild. Especially in patient 1, prompt cessation of MMI treatment was required to prevent further progression of polyarthritis.

The reported frequency of appearance of MPO-ANCA caused by ATDs ranges from 4.1% to 64% (1,4,5). MPO-ANCA vasculitis caused by PTU is more common than that resulting from MMI (4,5,12,13,14). Noh et al (8) reported that while the incidence of MPO-ANCA vasculitis caused by PTU was 39.2 times higher than that by MMI, one 14-year-old patient had MMI-related MPO-ANCA involving the kidneys and joints. In addition, several pediatric cases with MPO-ANCA vasculitis caused by MMI have been reported (6,7). Accordingly, it is important to distinguish polyarthritis as a side effect of MMI from MPO-ANCA vasculitis.

It has been reported that larger doses are associated with increased frequencies of all side effects, including polyarthritis (4,5). Ploegstra et al (9) reported a 15-year-old girl with arthritis caused by MMI. At the onset of her arthritis, the dose of MMI was 90 mg/day, prompting the authors to suggest that the high dose of MMI might have been related to the development of arthritis. According to the American Thyroid Association (ATA) guideline, the dose of MMI typically used is 0.2-0.5 mg/kg daily, with a range from 0.1 to 1.0 mg/kg daily (1). One approach is to prescribe 10-20 mg/day in 10-18 years old patients with GD. With severe clinical and biochemical hyperthyroidism, doses that are 50-100% higher than this dose can be used (1). In our patient 2, the MMI dose was 50 mg (1.25 mg/kg/day), which exceeded the dose recommended for children by the ATA guideline. The dose of MMI in patient 1 was 30 mg (0.5 mg/kg/day), however, this dose was used as an initial dose. Therefore, the starting dose should be lower than this to avoid side effects.

The onset of polyarthritis in our cases and in a case reported by Ploegstra et al (9) occurred within one month after the initiation of MMI. These findings indicate that arthritis caused by MMI warrants attention, especially when it occurs within one month after the initiation of MMI.

In conclusion, these cases highlight the need for greater awareness of this relatively rare adverse effect of MMI. Prompt cessation of MMI may be required in such cases.
